# Contribution of H-Bonding to the Preference of Platinum Anti-Tumour Drugs for Particular Bases and Particular Cross-Links

**DOI:** 10.1155/MBD.1994.311

**Published:** 1994

**Authors:** Giovanni Natile

**Affiliations:** 1 Dipartimento Farmaco-Chimico via E. Orabona 4 Bari I-70125 Italy

## Abstract

The stereochemical factors that influence the tendencies for sequence specific binding of platinum antitumour drugs to DNA are examined. The NHs of the platinum-amine moiety can form hydrogen bonds to the O6 of guanine or to a phosphate oxygen of DNA. Modelling the stereochemistry of the NH atoms can lead to compounds with a strong preference for forming one type of adduct with DNA.


### CONTRIBUTION OF H-BONDING TO THE PREFERENCE OF PLATINUM ANTI-TUMOUR DRUGS FOR PARTICULAR BASES AND PARTICULAR CROSS-LINKS

Giovanni Natile

Dipartimento Farmaco-Chimico, via E. Orabona 4, I-70125 Bari, Italy

**Abstract**

The stereochemical factors that influence the tendencies for sequence specific binding of platinum antitumour drugs to DNA are examined. The NHs of the platinum-amine moiety can form hydrogen bonds to the O6 of guanine or to a phosphate oxygen of DNA. Modelling the stereochemistry of the NH atoms can lead to compounds with a strong preference for forming one type of adduct with DNA.

### Introduction

Anti-cancer compounds such as *cis*-$[Pt(NH_3)_2Cl_2]$ (*cis*-DDP) and $[Pt(en)Cl_2]$ preferentially attack purine moieties in DNA [1], the most common point of attack being the N7 of guanine [2]. The nature of the non-leaving groups, i.e. the amine ligands, plays an important, but not completely defined role in the anti-cancer activity of the drug. The requirement that at least one hydrogen be attached to each nitrogen for significant anticancer activity [3] has led to the speculation that this NH group forms a hydrogen bond to the O6 of guanine [4] or to a phosphate oxygen [5].

In DNA, *cis*-platinum compounds crosslink adjacent purine residues [2] in a Head-to-Head (HH) conformation in which both the H8 atoms are on the same side of the platinum coordination plane [6]. The bifunctional adducts fall into two classes: (i) intrastrand, i. e. d(GpG) (60%), d(ApG) (25%) and d(GpNpG) (<10%) and (ii) interstrand linking two guanines (<10%) [7].

There is also evidence that platinum compounds form monofunctional adducts with DNA which are precursors of the bifunctional adducts. The factors that influence the frequency of the monofunctional adducts will indirectly affect the frequency of the bifunctional adducts. Also, the geometry of the monofunctional adduct may influence the propensity for formation of a bifunctional adduct.

This article concentrates on the contribution of H-bonding to the preference of platinum for particular bases and particular cross-links.

### Stereochemical control in Pt-DNA monofunctional binding

Formation of the monofunctional adducts requires that one of the chloro groups of *cis*-DDP be displaced. It is believed that, on entering the cell, *cis*-DDP is hydrolyzed, resulting in replacement of one or both chloride ligands by water and/or hydroxyl ligands [8].

Under neutral conditions, platinum binds to the N7 atom of guanine, the N7 and N1 atoms of adenine, and the N3 atom of cytosine. In DNA, atoms involved in base pairing, i.e. N1 of adenine and N3 of cytosine, are less available for metal binding than N7 atoms of guanine and adenine which are exposed in the major groove. When platinum binds to N7 of guanine or adenine, it is forced into close proximity with the substituent in the 6-position of the purine. In the case of guanine, this substituent is an O(carbonyl) atom and Pt···O contacts in the range 3.28-3.53 Å are observed. In the case of adenine, the substituent is an $NH_2$ group (Figure 1). The interaction with the $NH_2$ group is almost certainly more destabilizing than that with the carbonyl oxygen and so contributes to the preference Pt(II) has for N7 of guanine over N7 of adenine. This preference in the binding of *cis*-DDP

to DNA was proved by the observation that only monofunctional adducts with N7 of guanine, and not N7 of adenine, are found on digestion of *cis*-DDP-treated DNA [9].

Figure 1. Diagram of *cis*-DDP coordinated to N7 of Adenine and Guanine in A-T and G-C base pairs, respectively.

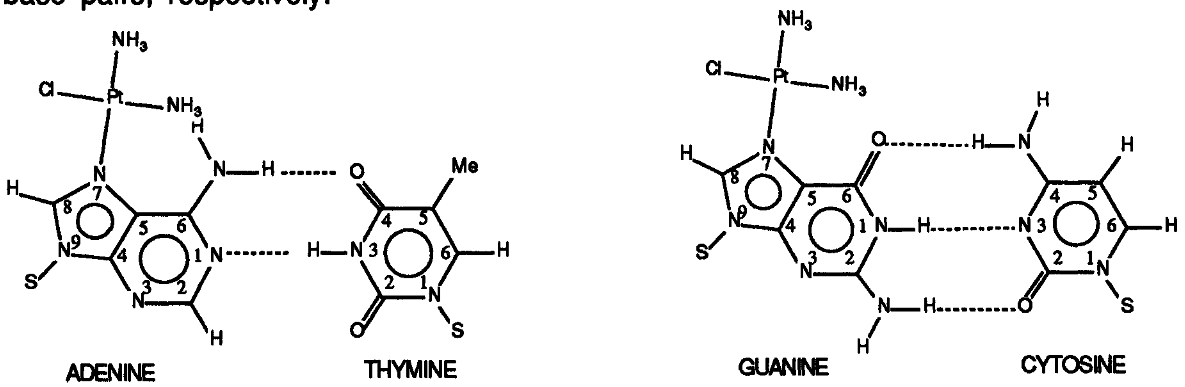

**Stereochemical control in Pt-DNA bifunctional binding.**

While there is as yet no unequivocal evidence that bifunctional binding of *cis*-DDP to adjacent purine bases is responsible for its cytotoxic activity, such interactions are unquestionably those which occur most frequently between *cis*-DDP and DNA and have consequently attracted the greatest amount of attention.

The major binding site was found to be adjacent guanine bases of one strand [d(GpG)] with a smaller amount bound to the d(ApG) sequence [9, 10]. Binding to d(GpA) sequences was not found, or occurred at very low frequency, in binding studies with DNA [9, 10] and the trinucleotide d(GpApG) [11].

The lower preference for d(ApG) binding compared with d(GpG) binding is understandable on the basis of the lower kinetic preference for binding to adenine compared with guanine [12]. However, this does not explain why binding does not occur to d(GpA) sequences. Dewan has pointed out that when *cis*-DDP binds to the central guanine in a d(ApGpA) sequence of B-DNA, the distance from the Pt atom to the N7 of the adenine on the 5'-side is ~3 Å and the distance to the N7 of the adenine on the 3'-side is ~5 Å [13]. This observation is consistent with a kinetic preference for the formation of d(ApG) adducts over d(GpA) adducts. However molecular mechanics analysis of monofunctional binding performed by Hambley showed that these distances change considerably; those on the 5'-side then range from 4.55 to 5.59 Å and those on the 3'-side from 3.86 to 5.25 Å. In nearly all cases, the distances were similar, with that on the 5'-side generally slightly longer than that on the 3'-side, which is the reverse of the situation seen in the idealized models [14].

The interaction between a $NH_3$ ligand and groups in the 6-position of the 3'-purine was proposed to contribute significantly to the difference in the frequencies of occurrence of the d(ApG) and d(GpA) adducts. Models of *cis*-DDP bound to d(GpG) sequences of A-DNA showed that there is a hydrogen bond between one ammine ligand and O6 of the guanine in 3'-position and another between the other ammine and a terminal oxygen of the 5'-phosphate group [15] (Figure 2). When bound to B-DNA, *cis*-DDP makes the former of these hydrogen bonds with equal facility but the orientation of the sugar-phosphate backbone is such that the second does not form. Rearrangement of the 5'-sugar ring from C2'-endo to C3'-endo brings the phosphate group into a position where the hydrogen bond can form, yielding a model similar to that seen previously for *cis*-DDP bound to A-DNA [5]. NMR studies have shown that formation of *cis*-DDP adducts generally causes a change in the sugar conformation from C2'-endo to C3'-endo [16]. The same interaction was observed in models of bifunctional binding and in the crystal structures of *cis*-DDP bound to d(pGpG) and d(CpGpG) [17].

Figure 2. Diagram of $[Pt(NH_3)_2\{d(pGpG)\}]$ showing the proximity of the ammine ligands to O6 of guanine in 3' position and to a terminal oxygen of the 5'-phosphate group respectively.

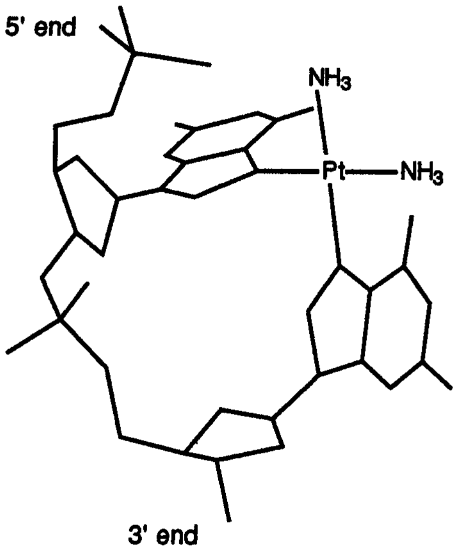

When *cis*-DDP binds to the d(ApG) sequence the situation is very similar to that described previously for *cis*-DDP bound to GpG sequences. In contrast when *cis*-DDP binds to the d(GpA) sequence of A-DNA or modified B-DNA (C3'-endo conformation of the 5' sugar), the hydrogen bond to the 5'-phosphate is observed as before. However, since the purine on the 3'-side is now adenine, which has an $NH_2$ group in the 6-position, no hydrogen bond between this purine and an ammine is possible. Rather, there are repulsive H···H contacts in the range 2.59-2.64 Å. This could explain the difference in the frequencies of occurrence of the d(ApG) and d(GpA) adducts.

The proposal that interactions between an ammine ligand and groups in the 6-position of the 3'-purine influence binding specificity was tested by designing compounds able to interact favourably with the $-NH_2$ group of an adenine in the 3'-position. Such a compound should be able to bind to d(GpA) sequences in preference to d(ApG) and d(GpG) sequences. One of these compounds was 2(*S*),S(*S*)-dichloro-methioninemethylester-sulfoxide-platinum(II) in which the O-atom of the sulfoxide is correctly oriented to H-bonding to the adenine $NH_2$ group [18].

**Modelling the stereochemistry of the NH atoms.**

An approach to the design of compounds which should have a strong preference for forming one type of adduct with DNA, is to model the stereochemistry of the N-H atoms of the non-leaving amine ligands so that they could bind stereospecifically to DNA [19].

The *cis*-dichloro(ethylenediamine)platinum(II) which has been extensively studied for its close similarity to *cis*-DDP and because it can be conveniently radiolabeled [9, 20] has a puckered chelate ring which can assume either λ or δ conformation. The rate of λ-δ interconversion is, however, fast under normal conditions and the average situation is that of a planar chelate ring with the two hydrogens at each aminic group equally displaced above and below the coordination plane [21].

Structurally related to ethylenediamine is 1,2-diaminocyclohexane (DACH). The organic chain bridging the two nitrogens has two chiral carbons and the DACH ligand exists in three isomeric forms with the *R,R*, *S,S*, and *R,S* configurations at the asymmetric centres. The puckering of the five-membered chelate ring is determined by the configuration of the ligand; is δ in the *S,S* isomer; λ in the *R,R* isomer and both λ and δ, rapidly interconverting, in the *S,R* isomer.

Kidani and coworkers reported that platinum complexes with DACH had biological activities depending upon the chirality of the diamine ligand. The *R,R* isomer was apparently endowed with greater antitumour activity and was less mutagenic than the *S,S* isomer [22].

The reaction of the sulphate form of each of these isomers with DNA was characterized as a possible explanation for the apparent differences in antitumour activity [23]. The spectrum of adducts produced was similar for each isomer and similar to that reported for *cis*-DDP with adduction at d(GpG), d(ApG) and $(dG)_2$. The kinetics of formation of the various adducts was the same for each isomer; total platination of DNA was complete in 15 min as were bifunctional adducts at d(GpG) and $(dG)_2$. However, rearrangement to bifunctional adducts took several hours in the case of adducts at d(ApG) sequences. These results did not provide a reason for the different activities of the isomers. It was suggested that the interaction of these adducts with metabolic processes such as DNA repair might explain the observed differences.

A comparative study of three platinum complexes with chiral diamines $[PtCl_2(N\text{-}N)]$ (N-N = 1,2-diaminopropane, DAP; 2,3-diaminobutane, DAB; and 1,2-diamino-cyclohexane, DACH) was also carried on [24, 25] (Figure 3).

Figure 3. Diagram of $[PtCl_2(DAP)]$, $[PtCl_2(DAB)]$ and $[PtCl_2(DACH)]$ complexes showing the configuration of the asymmetric carbons and the puckering of the chelate rings.

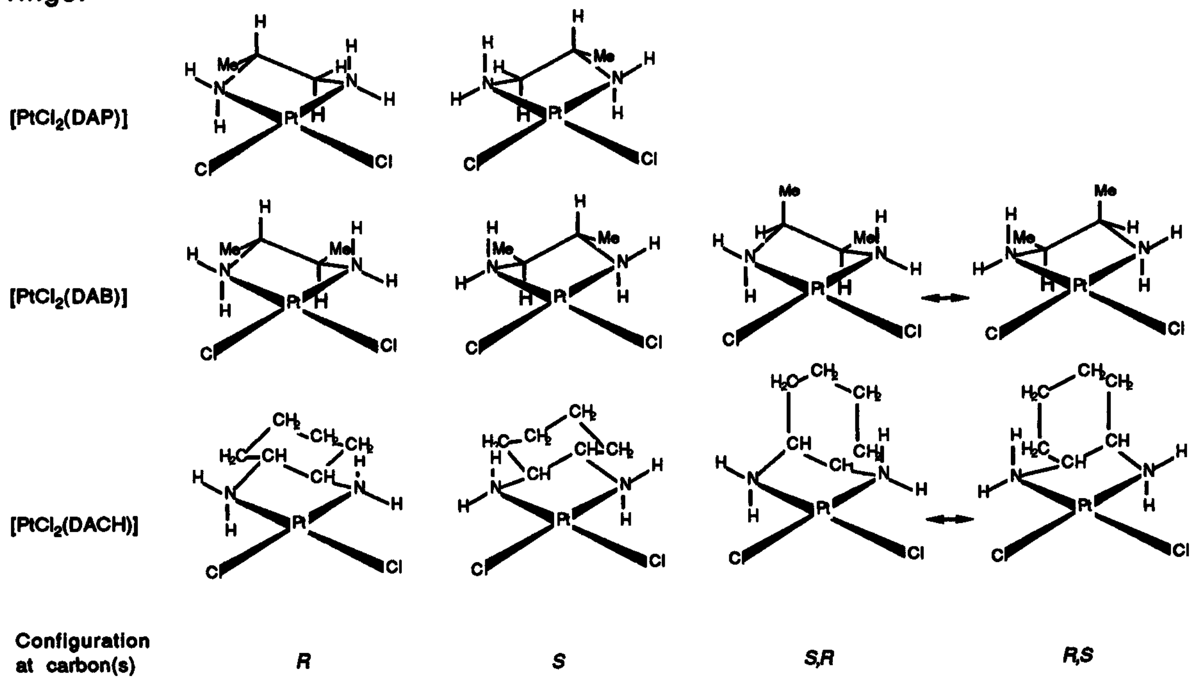

The biological tests, *in vitro*, revealed a marked difference among isomers. For instance the mutagenic activity, which is strictly related to the interaction of the drug with DNA, could be even ten times greater in one isomer with respect to the corresponding enantiomer. In all cases examined the *S,S* isomer was by far the most mutagenic indicating that the different isomers give adducts with DNA which can be discriminated by the enzymatic systems involved in mutagenesis. The most striking differences being observed for the DAB complexes.

Molecular models indicate that the stereochemistry of the N-hydrogens in the *S,S* isomer is such to favour O6-NH hydrogen bonding of the guanine in the 3'-position (a

*quasi-axial* hydrogen on the same side of O6 with respect to the platinum coordination plane). This interaction, together with the hydrogen bonding of the second ammine to the 5'-phosphate group is known to stabilize the bifunctional binding of *cis*-DDP to d(GpG) and d(ApG) sequences. [It is to be noted that the DAB species can interconvert the δ and λ conformation of the chelate ring, however the preference for the equatorial position of the methyl substituents makes the δ conformation favoured when the configuration of the asymmetric carbons is *S* and the λ conformation favoured when the configurations of the asymmetric carbons is *R*].

**Stereochemical control in Pt-mononucleotide interaction.**

The ability of platinum compounds with chiral diamines to bind stereospecifically could also be proved in reactions with mono-nucleotides. In DNA *cis*-DDP cross-links adjacent purine residues in a Head-to-Head (HH) conformation in which both the H8 atoms are on the same side of the platinum coordination plane. When the two nucleotide moieties are not linked by a phosphodiester group, the purines can have orientations in which the H8s are on opposite sides of the platinum coordination plane; this orientation is designated Head-to-Tail (HT) [26]. Normally only HT complexes are detected in solution and in most solid state crystallographic studies [27, 28]. The HH atropisomer, which is the best model for intrastrand binding of *cis*-platinum compounds to DNA, is difficult to isolate and has been found in only a few crystal structures of *cis*-$[Pt(NH_3)_2$(9-ethylguanine)$_2]X_2$ [29].

A recent investigation of complexes of the type [Pt(5'GMP)$_2$(*R,S,S,R*-Me$_2$DAB)] [Me$_2$DAB = N,N'-dimethyl-2,3-diaminobutane and the configurations at the four asymmetric centres are *R, S, S,* and *R* at N, C, C, and N, respectively] led to the first evidence for the existence of a HH atropisomer in solution [30] (Figure 4). In addition, the HH atropisomer was found to exist in equilibrium with two HT atropisomers, the predominant HT having the Λ conformation. This was the first determination of the chirality of a HT species in solution; the ΔHT conformation had been found in all documented solid state structures for 6-oxopurine nucleot(s)ide complexes with metal centres [28, 31].

The favoured HT atropisomer could form two O6-NH H-bonds, the HH atropisomer could form one O6-NH H-bond and the other HT atropisomer could not form any O6-NH H-bond. Thus, O6-NH H-bond appeared to dominate the stereochemistry of these complexes. For the complex with all asymmetric centres on Me$_2$DAB inverted, [Pt(5'GMP)$_2$(*S,R,R,S*-Me$_2$DAB)], the dominant atropisomer had the ΔHT configuration. This result demonstrated that the stereochemistry of the ammine ligand influences the conformational equilibrium between atropisomers. The use of platinum(II) complexes of stereochemically controlling ligands shows promise for controlling DNA or RNA conformations.

A similar investigation performed on the less symmetrical [Pt(5'GMP)$_2$-(*S,R,R,R*-Me$_2$DAB)] and [Pt(5'GMP)$_2$(*S,S,S,R*-Me$_2$DAB)] species has also demonstrated that both O6-NH and ROPO$_3$-NH H-bonds are important in determining the atropisomer formed [32].

The O6 can participate in H-bonding only when the O6 and the NH are on the same side of the coordination plane. When, instead, the H8 and the NH are on the same side, only phosphate group H-bonding is possible since in the normal anti conformation of 5'GMP, the phosphate group is close to H8 and, thus, NH. Because the sugar moiety is flexible and there is relatively free rotation about the glycosyl bond, it is possible that a H-bond between the phosphate group and the NH is formed also when the H8 and NH are on opposite sides of the coordination plane. A quasi-equatorial NH was found to be particularly suitable to donate a strong hydrogen bond to the phosphate.

On the basis of molecular mechanics calculations it has been hypothesized that a diaminedichloroplatinum(II) complex having N-hydrogens lying in the platinum-coordination plane would hydrogen bond preferentially phosphate groups of opposite strands and bind interstrand in preference to intrastrand. Such a compound has been designed and prepared, and the results of cross-linking, DNA unwinding, and enzymatic digestion/HPLC studies of platinated DNA of one such compound, namely $[PtCl_2(HPIP)]$ (HPIP = 1,4-aza-cycloheptane), were consistent with the prediction [33].

Figure 4. Diagram of different atropisomers for $[Pt(5'GMP)_2(R,S,S,R\text{-}Me_2DAB)]$

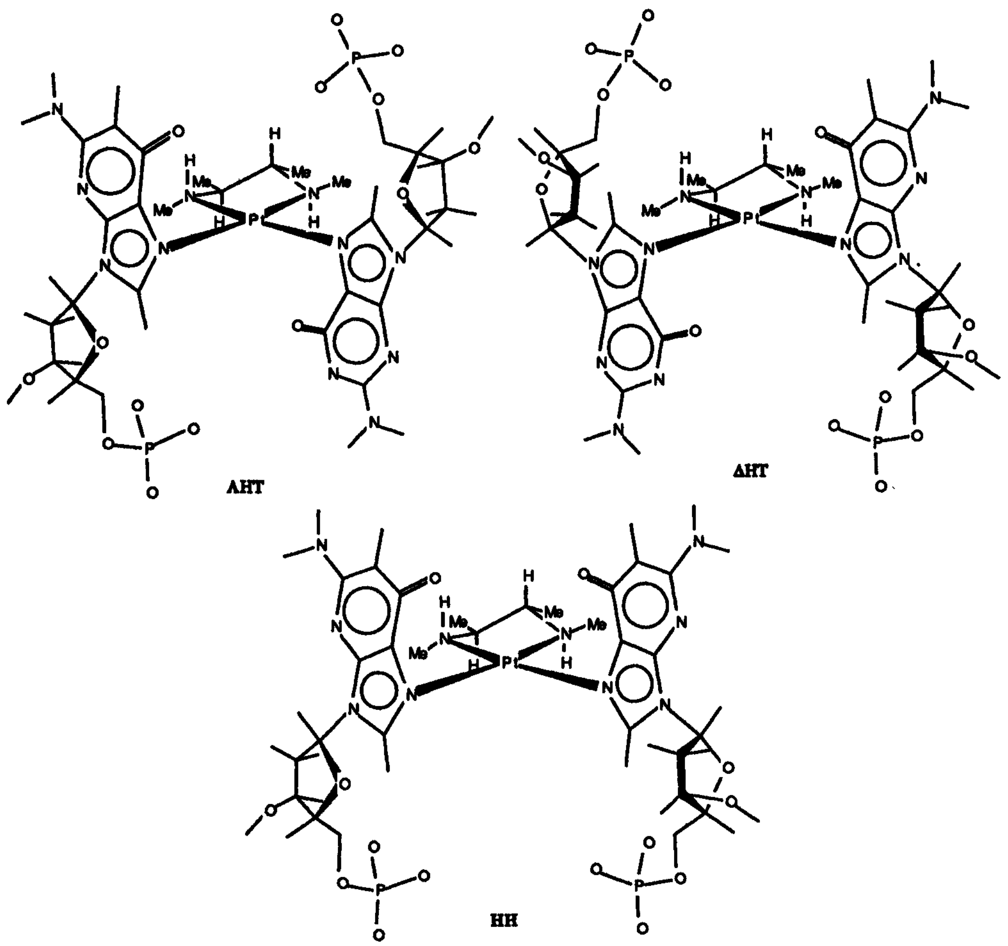

**Stereochemical control in platinum complexes with *trans* geometry.**

*Trans*-DDP reacts extensively with DNA [34], however is sterically restricted in the type of intrastrand cross-links it can feasibly produce; that is, it is unable to cross-link neighbouring bases in DNA. Such cross-links represent about 90% of the adducts produced by *cis*-DDP.

After a 1-h incubation, 85% of *trans*-DDP binds to double-stranded DNA as monofunctional adducts of deoxyguanosine (dG) and only 10% of the platination results as bifunctional adducts within the first few hours of reaction. These cross-links are between two dGs. Rearrangement to bifunctional adducts was only 50% complete in 24 h and 80% in 48 h [35]. A quantitation of the distribution of Pt in DNA modified at 1:100 Pt/nucleotide showed that dG-Pt-dC, dG-Pt-dG and dG-Pt-dA formed at approximately 50%, 40% and 10% of the bifunctional adducts, respectively. The equivalent values for the bifunctional adducts formed in single stranded DNA were dG-Pt-dG, 60%; dG-Pt-dA, 35%; and dG-Pt-dC, 5% [36]. Because the dG-Pt-dC bifunctional adducts were more prevalent in double-stranded DNA (40%) than in single stranded DNA (5%), they may derive from interstrand cross-links [37]. The remaining bifunctional adducts may derive from intrastrand cross-links between two bases separated by one or more intermediate bases. In general, much larger doses of *trans*-DDP than *cis*-DDP are required to form an equal number of platinum adducts on DNA [38].

More recently has been reported that the presence of a planar ligand, such as pyridine, greatly enhances the cytotoxicity of the *trans* structure, such that cytotoxicity is equivalent to that of the analogous *cis*-isomer and, indeed, *cis*-DDP itself [39]. Intracellular uptake was found to be enhanced for pyridine complexes relative to ammine complexes although binding to calf thymus DNA was significantly less for pyridine than for the analogous ammine complexes [40].

Also substitution of iminoether for ammine in *cis*- and *trans*-DDP has been found to reverse the biological activity of the *cis*- with respect to the *trans*-isomer (Figure 5). The complex with trans geometry showed the greatest in vitro cytotoxicity against P388 leukemia cells and displayed a relevant antitumour activity on P388 and P388/DDP-bearing mice [41].

Figure 5. Diagram of *trans*- and *cis*-$[PtCl_2\{NH{=}C(OMe)Me\}_2]$ complexes

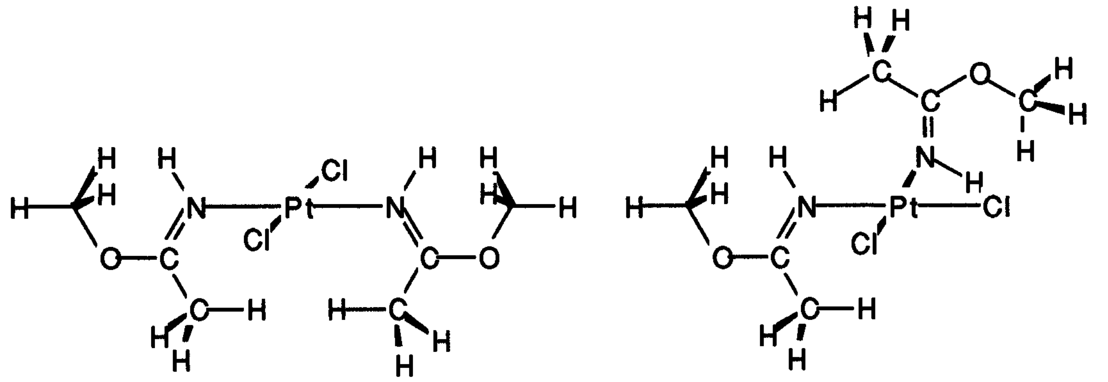

The *trans*-iminoether complexes are characterized by a remarkable inability to give interstrand cross-links which, instead, are formed in the reaction of *trans*-DDP with double stranded DNA [42]. Again hydrogen-bonding might play a key role. The N-bound hydrogen of the iminoether ligand is shielded by the ketoether residue and certainly is in an unfavourable position to hydrogen bond phosphodiester residues of opposite strands, a type of interaction which appears to promote the interstrand cross-links formation.

### Conclusion

It appears that by varying the stereochemistry of the N-Hs and the H-bonding ability of the non-leaving ligands, it is possible to determine the preference of platinum complexes for forming one type of adduct. If one of these lesions were responsible for antitumour activity, it could be possible to produce platinum drugs with the maximum possible activity.

### Acknowledgement

Support by the Ministero dell'Università e della Ricerca Scientifica e Tecnologica (quota 40%) is gratefully acknowledged.

### References

[1] S. Mansey, G. Y. H. Chu, R. E. Duncun, R. S. Tobias, *J. Am. Chem. Soc.* **100** (1978) 607. S. E. Sherman, S. J. Lippard, *Chem. Rev.* **87** (1987) 1153. J. Reedijk, *Pure Appl. Chem.* **59** (1987) 181. C. A. Lepre, S. J. Lippard, *Nucleic Acids and Molecular Biology*, F. Eckstein and D. M. J. Lilley (Ed.); Vol **4**, Springer-Verlag, Berlin, 1990 p. 9.

[2] A. M. J. Fichtinger-Schepman, J. L. van der Veer, J. H. J. den Hartog, P. H. M. Lohman, J. Reedijk, *Biochemistry*, 24 (1985) 707. K. Inagaki, K. Kasuya, Y. Kidani, *Chem. Lett.* (1984) 171. A. M. J. Fichtinger-Schepman, A. T. van Oosterom, P. H. M. Lohman, F. Berends, *Cancer Res.* **47** (1987) 3000.

[3] F. K. V. Leh, W. Wolf, *J. Pharm. Sci.* **65** (1976) 315. J. Reedijk, A. M. J. Fichtinger-Schepman, A. T. van Oosterom, P. van de Putte, *Struct. Bonding (Berlin)*, **67** (1987) 53. N. P. Johnson, J. -L. Butour, G. Villani, F. L. Wimmer, M. Defais, V. Pierson, V. Brabec, *Prog. Clin. Biochem. Med.* , **10** (1989) 1.

[4] T. W. Hambley, *Inorg. Chem.* **27** (1988) 1073.

[5] J. Kozelka, G. A. Petsko, S. J. Lippard, G. J. Quigley, *J. Am. Chem. Soc.* , **107** (1985) 4079. J. Kozelka, G. A. Petsko, G. J. Quigley, S. J. Lippard, *Inorg. Chem.*, **25** (1986) 1075. S. J. Berners-Price, U. Frey, J. D. Ranford, P. J. Sadler, *J. Am. Chem. Soc.*, **115** (1993) 8649.

[6] J. P. Caradonna, S. J. Lippard, *Inorg. Chem.* **27** (1988) 1454, and references cited therein. J.-M. Neumann, S. Tran-Dinh, J. -P. Girault, J. C. Chottard, T. Huynh-Dinh, J. Igolen, *Eur. J. Biochem.* **141** (1984) 465. T. P. Kline, L. G. Marzilli, D. Live, G. Zon, *J. Am. Chem. Soc.* **111** (1989) 7057 and references cited therein. J. H. H. den Hartog, C. Altona, J. H. van Boom, G. A. van der Marel, C. A. G. Haasnoot, J. Reedijk, *J. Biomol. Struct. Dyn.* **2** (1985) 1137. G. Admiraal, J. G. van der Veer, R. A. de Graaff, J. H. J. den Hartog, J. Reedijk, *J. Am. Chem. Soc.* **109** (1987) 592. S. E. Sherman, D. Gibson, A. H. -J. Wang, S. J. Lippard, *J. Am. Chem. Soc.* **110** (1988) 7368.

[7] A. Eastman, *Biochemistry*, **22** (1983) 3120.

[8] A. F. Le Roy, *Cancer Treat. Rep.* **63** (1979) 231.

[9] A. Eastman, *Biochemistry*, **22** (1983) 3927.

[10] A. M. J. Fichtinger-Schepman, J. L. van der Veer, J. H. J. den Hartog, P. H. M. Lohman, J. Reedijk, *Biochemistry*, **24** (1985) 707.

[11] J. L. van der Veer, H. van den Elst, J. H. J. den Hartog, A. M. J. Fichtinger-Schepman, J. Reedijk, *Inorg. Chem.* **25** (1986) 4657.

[12] R. B. Martin, *Acc. Chem. Res.* **18** (1985 ) 32. S. -H. Kim, R. B. Martin, *Inorg. Chim. Acta*, **91** (1984) 11.

[13] J. C. Dewan, *J. Am. Chem. Soc.* **106** (1984) 7239.

[14] T. W. Hambley, *Inorg. Chem.* **30** (1991) 937.

[15] T. W. Hambley, *Inorg. Chim. Acta*, **137** (1987) 15. T. W. Hambley, *Drug Des. Delivery*, **3** (1988) 153.

[16] J. H. J. den Hartog, C. Altona, J. -C. Chottard, J. -P. Girault, J. Y. Lallemand, F. A. A. W. de Leeuw, A. T. M. Marcelis, J. Reedijk, *Nuceic Acids Res.* **10** (1982) 4715.

[17] S. E. Sherman, D. Gibson, A. H. -J. Wang, S. J. Lippard, *Science*, **230** (1985) 412. G. Admiral, J. L. van der Veer, R. A. G. de Graaff, J. H. J. den Hartog, J. Reedijk, *J. Am. Chem. Soc.* **109** (1987) 592.

[18] E. C. H. Ling, G. W. Allen, T. W. Hambley, *J. Chem. Soc., Dalton Trans.* (1993) 3705.

[19] M. Coluccia, M. Correale, D. Giordano, M. A. Mariggiò, S. Moscelli, F. P. Fanizzi, G. Natile, L. Maresca, *Inorg. Chim. Acta*, **123** (1986) 225. M. Coluccia, F. P. Fanizzi, G. Giannini, D. Giordano, F. P. Intini, G. Lacidogna, F. Loseto, M. A. Mariggiò, A. Nassi, G. Natile, *Anticancer Res.* **11** (1991) 281.

[20] A. Eastman, *Biochemistry*, **25** (1986) 3912.

[21] G. Giannini and G. Natile, *Inorg. Chem.* **30** (1991) 2853 and references therein.
[22] Y. Kidani, K. Inagaki, R. Saito, S. Tsukagoshi, *J. Clin. Hematol. Oncol.* **7** (1977) 197.
[23] M. M. Jennerwein, A. Eastman, A. Khokhar, *Chem. -Biol. Interactions*, **70** (1989) 39.
[24] F. P. Fanizzi, F. P. Intini, L. Maresca, G. Natile, R. Quaranta, M. Coluccia, L. Dibari, D. Giordano, M.A. Mariggiò, *Inorg. Chim. Acta*, **137** (1987) 45.
[25] G. Natile and M. Coluccia, *'Chemistry and Properties of Biomolecular Systems'*, N. Russo, J. Anastassopoulou and G. Barone (Ed.), Vol. **2**, Kluwer, Dordrecht, 1994 p. 363.
[26] R. E. Cramer, P. L. Dahlstrom, *Inorg. Chem.* **24** (1985) 3420.
[27] M. D. Reily, L. G. Marzilli, *J. Am. Chem. Soc.* **108** (1986,) 6785.
[28] B. Lippert, *Prog. Inorg. Chem.* **37** (1989) 1.
[29] B. Lippert, G. Raudaschl-Sieber, C. J. L. Lock, P. Pilon, *Inorg. Chim. Acta*, **93** (1984) 43. G. Schöllhorn, G. Raudaschl-Sieber, G. Müller, U. Thewalt, B. Lippert, *J. Am. Chem. Soc.* **107** (1985) 5932.
[30] Y. Xu, G. Natile, F. P. Intini, L. G. Marzilli, *J. Am. Chem. Soc.* **112** (1990) 8177.
[31] One abstract of an incompletely refined structure contains a ΛHT atropisomer: R. Bau, R. W. Gellert, *Biochimie* **60** (1978) 1040.
[32] D. Kaiser, F. P. Intini, L. G. Marzilli, G. Natile, Y. Xu, unpublished work
[33] T. W. Hambley, G. W. Allen, R. R. Fenton, E. C. H. Ling, H. M. Er, K. Patser, *J. Inorg. Biochem.* **51** (1993) 559.
[34] L. A. Zwelling, T. Anderson, K. W. Kohn, *Cancer Res.* **39** (1979) 365. A. C. M. Plooy, M. van Dijk, P. H. M. Lohman, *Cancer Res.* **44** (1984) 2043.
[35] A. Eastman, M. A. Barry, *Biochemistry*, **36** (1987) 3303.
[36] A. Eastman, M. M. Jennerwein, D. L. Nagel, *Chem. Biol. Interact.* **67** (1988) 71.
[37] V. Brabec, M. Leng, *Proc. Natl. Acad. Sci. USA*, **90**, (1993) 5345.
[38] R. B. Ciccarelli, M. J. Solomon, A. Varshavsky, S. J. Lippard, *Bochemistry*, **24** (1985) 7533.
[39] M. Van Beusichem, N. Farrell, *Inorg. Chem.* **31** (1992) 634.
[40] N. Farrell, L. R. Kelland, J. D. Roberts, M. Van Beusichem, *Cancer Res.* **52** (1992) 5065.
[41] M. Coluccia, A. Nassi, F. Loseto, A. Boccarelli, M. A. Mariggiò, D. Giordano, F. P. Intini, P. Caputo, G. Natile, *J. Med. Chem.* **36** (1993) 510.
[42] M. Coluccia, M. A. Mariggiò, A. Boccarelli, F. Loseto, N. Cardellicchio, P. Caputo, F. P. Intini, G. Natile, unpublished work.

**Received: March 11, 1994 - Accepted: April 29, 1994**

